# Correlation Analysis between GDM and Gut Microbial Composition in Late Pregnancy

**DOI:** 10.1155/2021/8892849

**Published:** 2021-02-02

**Authors:** Genxia Li, Pan Yin, Shuhui Chu, Wanli Gao, Shihong Cui, Shuhua Guo, Yajuan Xu, Enwu Yuan, Texuan Zhu, Jie You, Junya Zhang, Manman Yang

**Affiliations:** ^1^Obstetrics Department, The Third Affiliated Hospital of Zhengzhou University, Zhengzhou 450052, China; ^2^Laboratory Department, The Third Affiliated Hospital of Zhengzhou University, Zhengzhou 450052, China; ^3^Nutrition Department, The Third Affiliated Hospital of Zhengzhou University, Zhengzhou 450052, China

## Abstract

The prevalence of GDM is very high worldwide. The specific pathogenesis of GDM is currently not very clear. Recent research suggests that changes in the intestinal flora during pregnancy play a key role in it. Therefore, this study is aimed at exploring the characteristics of the intestinal flora of patients with gestational diabetes in the third trimester of pregnancy and at finding the intestinal flora with significant differences in healthy pregnant women to provide a basis for future clinical attempts of using intestinal microecological agents to treat gestational diabetes mellitus (GDM). We sequenced the V3-V4 regions of the 16S ribosomal ribonucleic acid (rRNA) gene from stool samples of 52 singleton pregnant women at >28 weeks of gestation. Our results showed that there were significant differences between the NOR group vs. GDM group and the G group vs. LG group among *Bacteroides*, *Firmicutes*, and *Firmicutes/Bacteroides.* At the species level, there were significant differences in the abundance of eight species in the NOR and GDM groups. Among them, the relative abundance of *Clostridium_spiroforme*, *Eubacterium_dolichum*, and *Ruminococcus_gnavus* was positively correlated with FBG, and *Pyramidobacter_piscolens* was negatively correlated with FBG, whereas there were significant differences in the abundance of five species in the G and LG groups. Functional analysis showed that there were differences in the biosynthesis and metabolism of polysaccharides, digestive system, classification, and degradation of the intestinal microbes between the NOR and GDM groups and between the G and LG groups. These results indicated that the gut microbes between GDM patients in the third trimester of pregnancy and healthy controls had essential characteristic changes and might be involved in the regulation of patients' blood glucose levels.

## 1. Introduction

GDM refers to the first occurrence of varying degrees of glucose metabolism abnormalities during pregnancy. It is a common complication of pregnancy [1, 2]. According to the International Diabetes Federation (IDF), one in six mothers (16.8%) of live births had some form of hyperglycemia during pregnancy, and 84% of them were diagnosed with GDM [3]. The incidence of GDM in Asian populations could be up to 20%. Although most GDM patients return to normal postpartum glucose metabolism, 20% of them continue to have impaired glucose tolerance or impaired fasting blood glucose (FBG) postpartum, and GDM patients have a significantly higher lifetime risk of type 2 diabetes, which is 7.4 times that of non-GDM patients [4]. Recently conducted research also found that independent of obesity or type 2 diabetes, GDM is also a risk factor for hypertension, metabolic syndrome, and cardiovascular disease [5–8]. These aspects not only have a serious impact on the short- and long-term health of mothers and babies but also continue to increase medical costs and significantly increase the social burden. Therefore, early diagnosis of GDM or early identification of high-risk populations is particularly important.

The specific pathogenesis of GDM is currently not very clear. Recent research suggests that changes in the intestinal flora during pregnancy play a key role in it [9, 10]. In 2007, the “Human Microbiome Project” was proposed. In recent years, with the emergence of metagenomics and metabolomics, more and more diseases have been demonstrated to be related to the intestinal flora [11–13]. The normal intestinal flora and the mucosal surface form a natural barrier, which plays an essential role in digestion and absorption, regulating immune function and preventing the invasion of pathogenic bacteria. Studies at home and abroad have shown that intestinal microflora disorders play a key role in the pathogenesis of obesity, type 2 diabetes, inflammatory bowel disease, liver disease, kidney injury, autism, and other diseases [14–18]. Pregnancy, especially in the third trimester, is a unique period. As the body's metabolic needs increase, the intestinal flora, as a “metabolic organ,” will also undergo adaptive changes [19, 20]. The characteristics of the intestinal flora in the third trimester of pregnancy and its correlation with the onset of GDM have become research hotspots.

In this study, we used 16S rRNA high-throughput sequencing technology to detect the intestinal bacteria of pregnant women with GDM and normal pregnant women at different stages of pregnancy (28–36 weeks and 36–41 weeks) and analyzed the different flora of the three groups of pregnant women. If a difference exists between the intestinal flora of normal pregnant women and that of patients with GDM, this would help us understand the pathogenesis of GDM and provide a theoretical basis for future treatment of gestational diabetes by supplementing probiotics. Therefore, the aim of this study was to investigate whether there are differences between the intestinal flora of normal pregnant women and those with GDM by using 16S rRNA high-throughput sequencing technology to determine the intestinal flora of normal pregnant women and those with GDM in the third trimester of pregnancy.

## 2. Materials and Methods

### 2.1. Study Population

From October 1, 2018, to December 30, 2019, 52 singleton pregnant women at more than 28 gestational weeks were recruited from the outpatient department of the Third Affiliated Hospital of Zhengzhou University. Of these, 23 had GDM and were further divided into a longer gestational week (LG group, *n* = 12) and shorter gestational week (G group, *n* = 11) based on whether or not the gestational age was greater than 36 weeks. The remaining 29 were normoglycemic pregnant women (NOR group). The inclusion criteria were as follows: pregnant women without prepregnancy diabetes, hypertension, serious damage to other organs, other metabolic diseases, other endocrine diseases, history of chronic diseases of the digestive tract, diarrhea, or other gastrointestinal diseases in the past 4 weeks; those who were taking antibiotics or intestinal probiotics in the past 4 weeks; and those with abnormal routine stool examination. We excluded those specimens with insufficient amounts of specimen retention or contamination during specimen conservation. The Medical Ethics Committee of the Third Affiliated Hospital of Zhengzhou University approved the present study, and all participants provided a written informed consent form.

### 2.2. Diagnostic Criteria for GDM

The diagnosis of GDM was made if any of the following criteria were met: (1) FBG ≥ 5.1 mmol/L, (2) 1 h blood glucose post 75 g oral glucose load ≥ 10.0 mmol/L, and (3) 2 blood glucose post 75 g oral glucose load ≥ 8.5 mmol/L [21].

### 2.3. Sample Collection

Stool samples (1 g) were collected before breakfast and put into sterile plastic tubes. Adopting a sampling box to keep samples at low temperatures during 0.5 h transport back to the lab, in a −80°C refrigerated storage, completes DNA extraction within 48 h.

### 2.4. Detection of Biochemical Indicators

We used the Hitachi 7600-020 automatic biochemical analyzer to detect FBG (hexokinase method detection), total cholesterol (enzyme colorimetric detection), triacylglycerol (TG, enzyme colorimetry detection), high-density lipoprotein cholesterol (catalase removal method detection), and low-density lipoprotein cholesterol (catalase removal method detection). The five test item reagents were all produced by Sichuan Mike Biological Technology Co. Ltd. All experimental processes were tested with high- and low-level quality control products to ensure the accuracy of test results.

### 2.5. Bacterial DNA Extraction and 16S rRNA Gene Sequencing

DNA extraction and 16S rRNA gene sequencing were conducted by the Wuhan Huada Gene Sequencing Center. The extraction of total bacterial DNA from stool samples was conducted according to the instructions of the BGI Stool Genome Extraction Kit. A microspectrophotometer was used to detect the concentration and purity of DNA. When the ratio of absorbance 260/280 is 1.7 to 1.9, the extracted DNA is considered usable. We designed 16S rRNA gene PCR primers using total DNA as a template to amplify the 16S rRNA V3-V4 hypervariable region. Subsequently, the Illumina Hiseq 2500 PE250 protocol was used for targeted amplicon sequencing. We used the Flash software to merge the original opposite sequences and divide them with tags. The minimum overlap was 15 bp, and the mismatch rate was <0.1.

### 2.6. OTU Clustering

The operational taxonomic unit (OTU) refers to a unified mark set artificially for a certain taxonomic unit (line, genus, species, grouping, etc.) to facilitate analysis in phylogeny or population genetics research. We used the software USEARCH (v7.0.1090) to cluster the spliced tags into OTUs and usually cluster tags with a similarity of more than 97% into one OTU. The abundance of OTU preliminarily illustrates the species richness of the sample.

### 2.7. OTU Species Annotation

After obtaining the representative sequence of OTU, we used RDP classifier (v2.2) software to compare the representative sequence of OTU with the Greengenes database for species annotation and set the confidence level at 0.8. The annotation results were filtered as follows: (1) remove OTUs without annotation results and (2) remove annotation results that do not belong to the species in the analysis project. For example, if the sample is made of 16S bacteria and OTU annotates Archaea, it will be removed.

### 2.8. Statistical Processing

We used software R (v3.1.1)'s VennDiagram package to generate Venn diagrams and OTU Core-Pan diagrams and R (v3.2.1)'s mixOmics package for OTU PLS-DA analysis. We used R (v3.1.1)'s gplots package to generate a species abundance heat map whose distance algorithm was Euclidean and whose clustering method was complete. GraPhlAn (https://huttenhower.sph.harvard.edu/graphlan) was used to generate a species composition map. R (v3.4.1)'s cluster and clusterSim packages were used for flora typing analysis. R (v3.2.1) was used to generate an alpha diversity box plot. R (v3.4.1)'s ggplot package was used to generate Beta diversity index box plots. LEfSe (https://huttenhower.sph.harvard.edu/galaxy/) was used to analyze the cluster diagram and the LDA diagram. We used R (3.4.1) and the Kruskal-Wallis test to screen the different species and used R (v3.4.1) and Picrust software to predict the function of the flora. Cytoscape was used to draw species network diagrams. SPSS 23.0 was used for statistical processing. Normally distributed measurement data were represented by *x* ± *s*, and count data were represented by “percentage (%) or rate.” The comparison between the two groups was conducted by a *t*-test. Pearson's correlation coefficient was used to assess the correlation between blood glucose and different flora. *P* < 0.05 was considered statistically significant.

## 3. Results

### 3.1. Analysis of General Conditions and Biochemical Indicators between the Two Groups

According to the statistical analysis of the collected general data and laboratory data, there was no significant difference in age and gestational age between the GDM and the NOR groups. The levels of BMI, FBG, 2 h blood glucose, HbA1c and TG, CHOL, and LDL in the GDM group were significantly higher than those in the NOR group, and the HDL level was significantly lower in the GDM group than in the NOR group (*P* < 0.01). The comparison between the G and LG groups showed that only the difference in gestational age between the two groups was significant (*P* < 0.01), and differences in other indicators were not significant. Details are found in Table 1.

### 3.2. OTU Sequence Diversity and Richness

After 52 samples were sequenced, a total of 866 OTUs were generated after clustering. The sequence values obtained after OTU clustering were analyzed, and the results were as follows. The average effective sequence obtained from the fecal flora of the NOR group was 74841, and 779 OTUs were obtained after merging with 97% similarity, and the sequencing coverage depth (coverage index) was 0.99932. The average effective sequence obtained from the fecal flora of the GDM group was 74846. After 97% similarity merging, 721 OTUs were obtained, and the sequencing coverage index was 0.99938. Additionally, the GDM group was classified as the G group when the pregnancy was less than 36 weeks. After 97% similarity, 608 OTUs were obtained, and the sequencing coverage index was 0.99934. The LG group was made of pregnant women at ≥36 weeks of pregnancy, and 563 OTUs were obtained after 97% similarity merging, and the sequencing coverage index was 0.99941. The sequencing depth was above 0.99, indicating that the probability of undetected sequences in the sample group was low.

**Figure 1:**
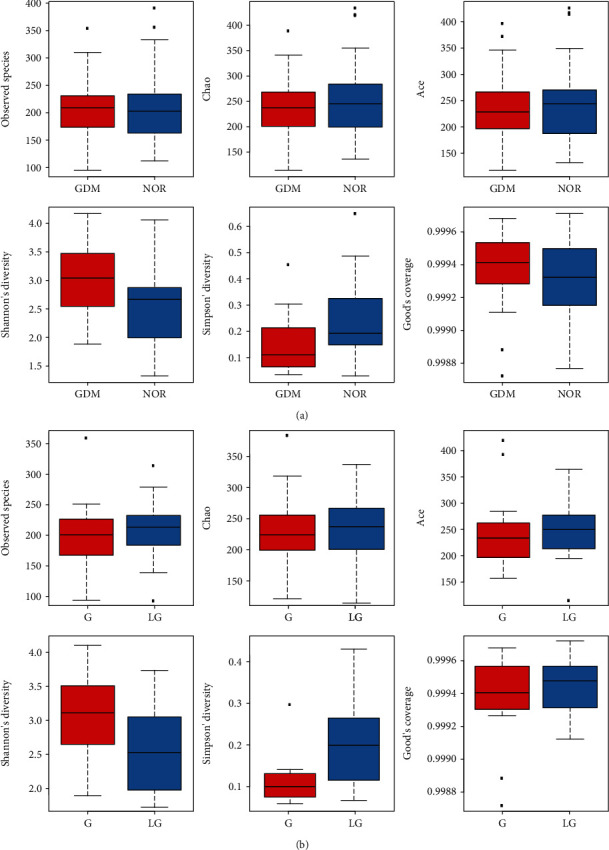
Analysis of OTU *α* diversity of intestinal flora in the NOR and GDM groups and the G and LG groups. (a) Comparison of the results of OTU *α* diversity analysis of intestinal microbes in the NOR and GDM groups. (b) Comparison of the results of OTU *α* diversity analysis of intestinal microbes in the G and LG groups.

The analysis of the alpha diversity index of the two groups of samples at the 97% similarity level showed that the observed species index, Chao index, Ace index, and coverage index levels of the GDM and NOR groups were similar, and there was no significant difference. The Shannon index of the GDM group was significantly higher than that of the NOR group, whereas the Simpson index was lower than that of the NOR group. It can be roughly estimated that the *α* diversity of the GDM group is greater than that of the NOR group. Additionally, among pregnant women with GDM, the fecal flora of gestation < 36 weeks (G group) and gestation ≥ 36 weeks (LG group) were analyzed by the alpha diversity index at the 97% similarity level. The average values of Chao and Ace indexes in the G group were 235 and 239, respectively, and in the LG group were 233 and 237. The results showed that the abundance of bacteria in the G group was higher than that in the LG group, but the difference was not statistically significant (*P* ≥ 0.05). The mean values of the Shannon and Simpson indices in group G were 3.21 and 0.10, respectively, and the mean values in the NOR group were 2.75 and 0.19, respectively. The diversity of flora in the G group was higher than that in the LG group, but the difference was not statistically significant (*P* ≥ 0.05). The specific results are shown in Figure 1.

**Figure 2:**
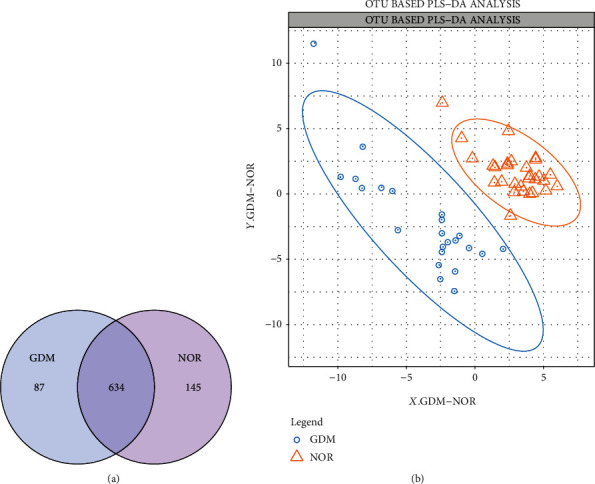
Comparison of the intestinal flora composition of the NOR and GDM groups. (a) Venn diagram showing the overlap of OTUs. (b) PLS-DA analysis of the difference in intestinal flora between the NOR and GDM groups.

A Venn diagram analysis showed that the NOR and GDM groups shared 634 OTUs (Figure 2(a)). PLS-DA analysis showed that the NOR and GDM groups were clustered and distinguished well (Figure 2(b)). It suggested that there were also significant differences in the composition and structure of the sample flora between the two groups.

To obtain the species classification information corresponding to each OTU, we used the RDP classifier Bayes algorithm to conduct taxonomic analysis on the OTU representative sequence and counted the bacterial composition of each sample at the level of phylum, class, order, family, genus, and species.

**Figure 3:**
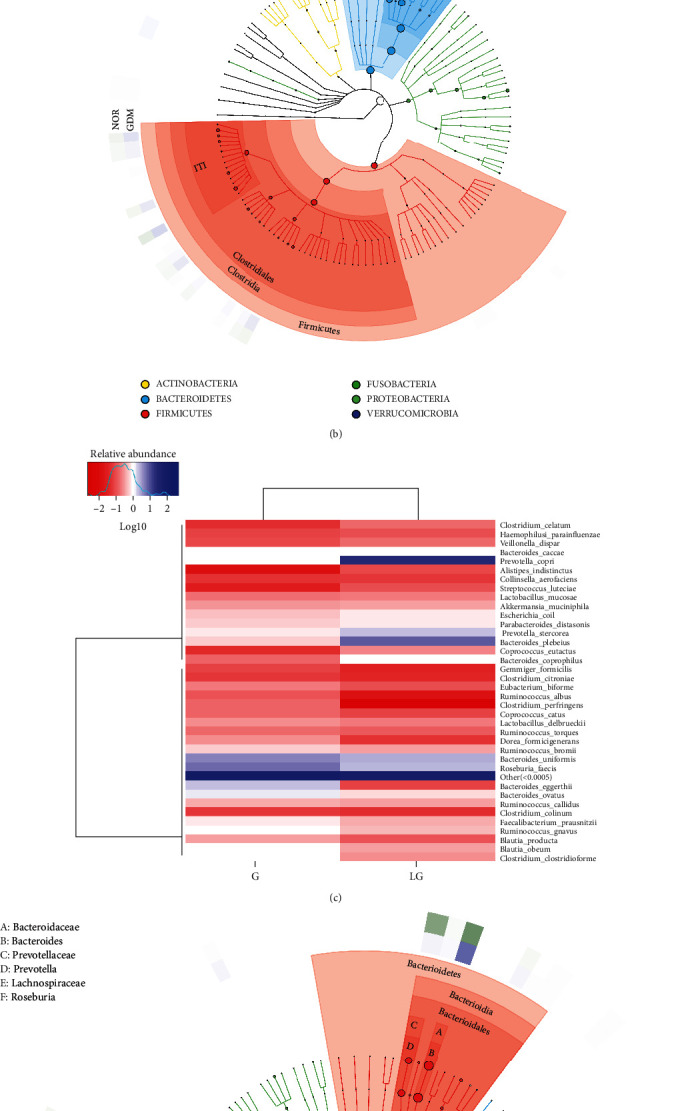
OTU species composition analysis. (a, c) NOR group, GDM group, G group, and LG group heat map cluster analysis. (b, d) NOR group, GDM group, G group, and LG group GraPhlan species composition analysis.

The heat map cluster analysis can visually display the clustering of samples from the same treatment or similar environment and reflect the similarity and difference in the bacterial composition of the samples (Figures 3(a) and 3(c)). The GraPhlan species composition map mainly displays the overall visual display of the species composition of each taxonomic level of the sample, distinguishes each taxum with different colors, reflects the species abundance of each taxonomy level through the size of the node, and uses the color depth of the outer ring heat map to represent the species abundance of each group. It can be used to discover dominant microbial groups (Figures 3(b) and 3(d)).

We analyzed the relative abundance of species in the GDM and NOR groups at the phylum level. In the NOR group, *Bacteroidetes*, *Firmicutes*, and *Proteobacteria* were the predominant flora, accounting for 68.2%, 23.2%, and 7.4%, respectively, of the total flora, whereas in the GDM group, accounting for 53.6%, 38.1%, and 5.1% of the total flora. The results showed that the proportion of *Bacteroides* in the GDM group was significantly lower than that in the NOR group, and the proportion of *Firmicutes* in the GDM group was significantly higher than that in the NOR group. The ratio of *Firmicutes*/*Bacteroides* in the GDM group (0.71) was significantly higher than that of the NOR group (0.34).

The dominant bacteria in the G and LG groups were Bacteroidetes (43.79% and 62.15%) and Firmicutes (48.29% and 29.19%). Bacteroidetes were significantly higher compared with the G group, and Firmicutes were significantly lower compared with the G group. The Firmicutes/Bacteroidetes ratio (1.10) of the G group was significantly higher than that of the LG group (0.47).

### 3.3. Analysis of Species Differences in Intestinal Microbiota

**Figure 4:**
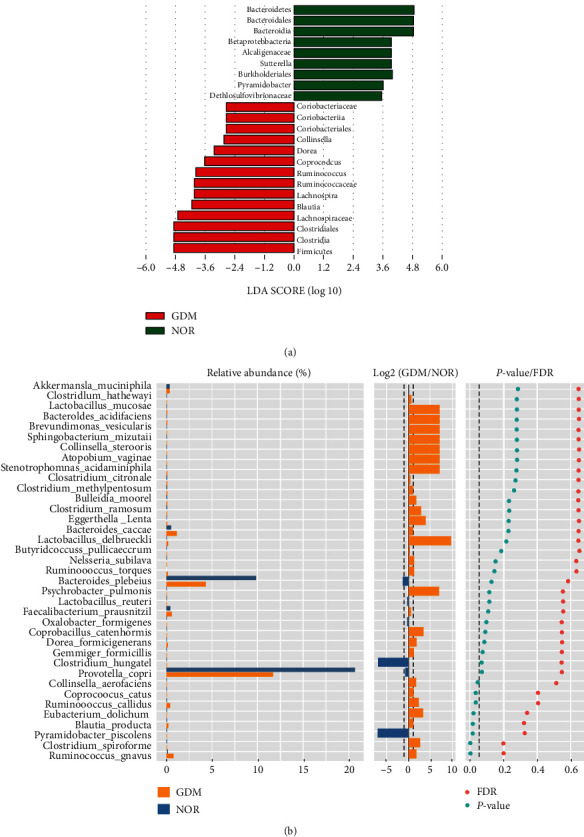
Analysis of the differences in intestinal flora species between the different groups. (a) LDA analysis of the NOR and GDM groups; (b) the NOR and GDM groups difference analysis of the Wilcoxon rank-sum test at the species level.

The LEfSe cluster analysis of the GDM group and the NOR group was conducted by LDA, and the results are shown in Figures 4(a) and 4(b). In the NOR group, nine groups of bacteria affected the difference between the groups, namely, *Bacteroidetes*, *Bacteroidales*, *Bacteroidia*, *Betaproteobacteria*, *Alcaligenaceae*, *Sutterella*, *Burkholderiales*, *Pyramidobacter*, and *Dethiosulfovibrionacea*. In the GDM group, the groups of bacteria that affected the difference between the groups were *Coriobacteriaceae*, *Coriobacteriia*, *Coriobacteriales*, *Collinsella*, *Dorea*, *Coprococcus*, *Ruminococcus*, *Ruminococcaceae*, *Lachnospira*, *Blautia*, *Lachnospiraceae*, *Clostridiales*, *Clostridia*, and *Firmicutes*.

Furthermore, through the Wilcoxon rank-sum test and the Mann-Whitney *U* test, the species of the two groups of samples in the phylum, class, order, family, genus, and species level for significant difference analysis and the difference in results at the species level are shown in Figures 4(a) and 4(b) and Table 2. The relative abundance of *Blautia producta*, *Clostridium spiroforme*, *Collinsella aerofaciens*, *Coprococcus catus*, *Eubacterium dolichum*, *Pyramidobacter piscolens*, *Ruminococcus callidus*, *Ruminococcus gnavus*, etc., differed significantly between the NOR and GDM groups. The above differences were statistically significant (*P* < 0.05).

### 3.4. Correlation Analysis of Intestinal Microbes and Blood Sugar

**Figure 5:**
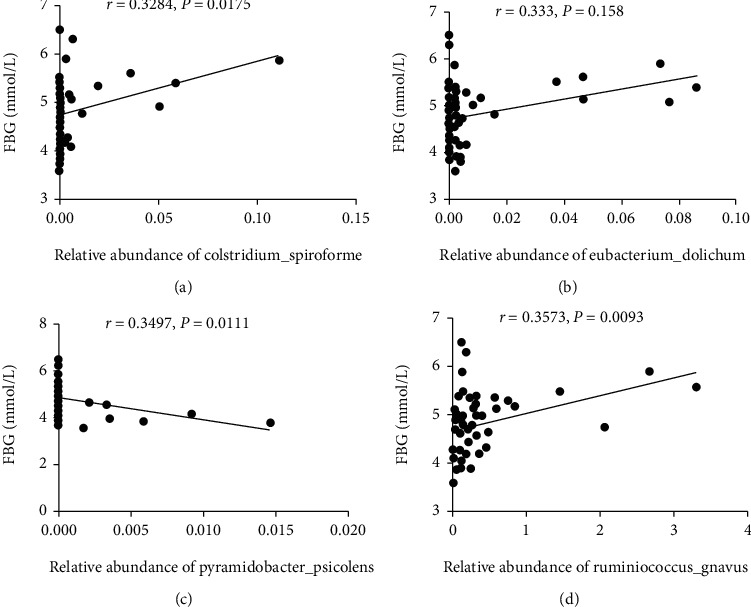
The correlation analysis between the relative abundance of gut bacteria with FBG.

To understand the close relationship between intestinal bacteria and blood glucose metabolism, the correlation between the relative abundance of the abovementioned different bacterial species and FBG was analyzed separately. The results are shown in Figure 5. The relative abundance of *Clostridium spiroforme* (*r* = 0.3284, *P* = 0.0175), *Eubacterium dolichum* (*r* = 0.3333, *P* = 0.0158), and *Ruminococcus gnavus* (*r* = 0.3573, *P* = 0.0093) in the NOR and GDM groups was positively correlated with FBG. *Pyramidobacter piscolens* (*r* = −0.3497, *P* = 0.0111) was negatively correlated with FBG. Other bacteria had no correlation with FBG.

### 3.5. Analysis of Differences in Intestinal Microbial Function

We calculated the abundance of each functional category based on the information in the KEGG database and the OTU abundance information. Additionally, for pathway, we used PICRUSt to obtain three levels of metabolic pathway information and also to obtain the abundance table of each level. Simultaneously, the 16S species information was mapped with the functional gene composition in the COG database to obtain the function prediction results. The COG database had two levels, namely, denoted cog_level1 and cog_level2.

**Figure 6:**
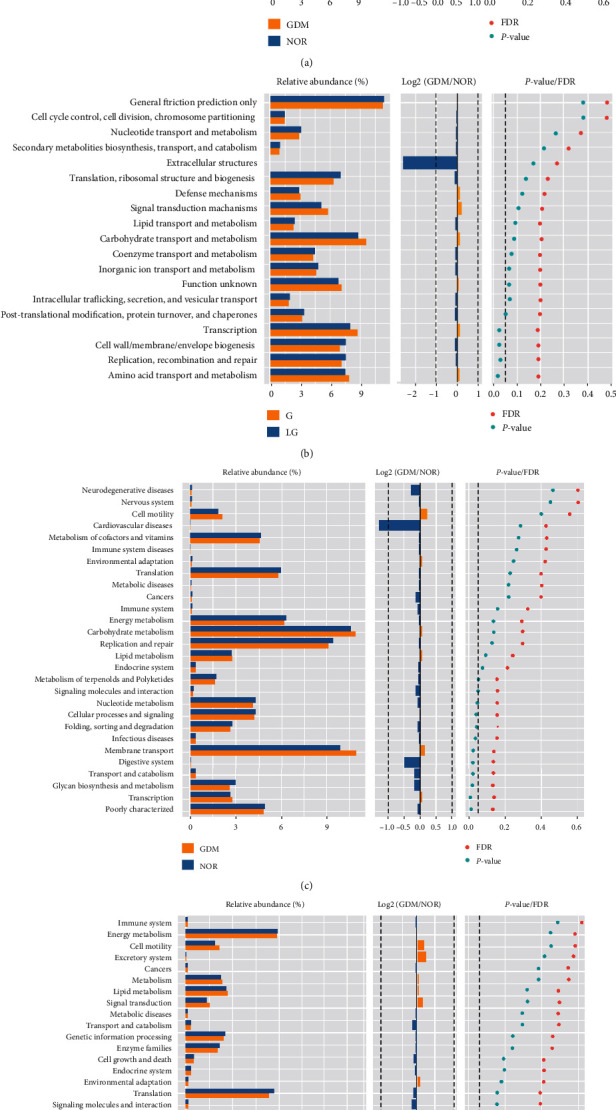
Analysis of the difference in intestinal microbial function between different groups. (a, b) Comparative analysis of COG level 2 difference between the NOR and GDM groups and the G and LG groups; (c, d) comparative analysis of KEGG level 2 difference between the NOR and GDM groups and the G and LG groups.

After we predicted the functions of all samples, we used the Wilcoxon test to find the difference function between each group. The comparison results of KEGG level2 and COG level2 of the NOR group vs. the GDM group and the G group vs. the LG group are shown in Figure 6. The results of the difference from the comparison of cog_level2 showed that the intestinal microbes of the NOR and GDM groups were significantly different in terms of cell wall/membrane/envelope biogenesis, organic ion transport and metabolism, posttranslational modification, protein turnover, and chaperones, transcription, function unknown, intracellular trafficking, secretion, and vesicular transport (Figure 6(a)). The gut microbes in the G and LG groups had significant differences in amino acid transport and metabolism, replication, recombination and repair, cell wall/membrane/envelope biogenesis, and transcription (Figure 6(b)). The kegg_level2 difference comparison results showed that the intestinal microbes of the NOR and GDM groups were significantly different in terms of poly characterized, transcription, glycan biosynthesis and metabolism, transport and catabolism, digestive system, membrane transport, infectious diseases, folding, sorting and degradation, cellular processes and signaling, nucleotide metabolism, and others (Figure 6(c)). The intestinal microbes of the G group and LG group were significantly different in terms of the digestive system, glycan biosynthesis and metabolism, infectious diseases, nucleotide metabolism, and metabolism of terpenoids and polyketides (Figure 6(d)).

## 4. Discussion

As a common metabolic disease in pregnant women, GDM seriously endangers the life, health, and safety of mothers and their offspring [22]. Studies have pointed out that changes in the intestinal flora might be related to the pathogenesis of GDM [23]. More and more scholars have noticed that changes in the structure of the intestinal flora could be the culprit in many metabolic diseases, such as obesity and type 2 diabetes, and gestational diabetes as a form of diabetes. In recent years, there has been more and more evidence that its onset could be related to the structural changes of the intestinal flora [24, 25]. Studies in China have found that compared with healthy pregnant women (mainly 24–28 weeks of gestation), the alpha diversity of gut microbiota of pregnant women with GDM of the same gestational age is reduced [26]. However, some studies have shown that the abundance of intestinal flora in pregnant women with GDM in the second trimester is higher than that of healthy pregnant women, but there is no significant difference in alpha diversity between the two groups [27]. This study showed that the alpha diversity of the intestinal flora of pregnant women with GDM in the third trimester was significantly higher than that of healthy pregnant women of the same gestational age. The difference between the three studies cannot be underlooked based on the gestational age. This shows that the current research on intestinal flora is still quite different, which might be related to factors such as differences in races, ages, dietary structure, living habits, experiments, and statistical methods. Generally, the study of intestinal flora and GDM lacks prospective studies with large samples and multiple regions, and scholars still need to continue exploring.

Additionally, the analysis of the relative abundance of species in the GDM and NOR groups showed that the dominant bacterial groups were *Bacteroidetes* and *Firmicutes*, but the proportion of *Bacteroidetes* in the GDM group was significantly lower than that in the NOR group, and the proportion of *Firmicutes* was significantly higher than that in the NOR group. The *Firmicutes/Bacteroidetes* ratio (0.71) of the GDM group was significantly higher than that of the NOR group (0.34). *Firmicutes* and *Bacteroidetes* are the two main dominant bacterial groups in the intestines. They can maintain the energy balance of the host by participating in the metabolism of fat and bile acids. Their ratio (*F*/*B* value) is often used as an indicator of the composition of the intestinal flora of different individuals [28–31]. A scholar named Ferrocino found that from the second to third trimesters of pregnancy, the number of *Firmicutes* in the intestine increased, whereas the number of *Bacteroides* and *Actinomycetes* decreased [32]. The increase in the number of *Firmicutes* can promote the metabolism of carbohydrates such as fructose, galactose, mannitol, starch, and sucrose in the intestines, thereby aggravating hyperglycemia, whereas the effect of *Bacteroidetes* is the opposite [33–36]. An imbalance of the intestinal flora in the second and third trimesters could affect the absorption of nitrogenous substances, block the bile acid cycle, affect the metabolism of sugar and fat in the body, and ultimately lead to GDM. Changes in the intestinal flora during pregnancy are similar to those of obese patients. In middle and late pregnancy, to meet the needs of fetal growth and development, pregnant women antagonize the increase in insulin-like substances in their bodies. The sensitivity of pregnant women to insulin decreases with increasing gestational age, and the insulin requirement increases accordingly. There is a certain degree of physiological insulin resistance (IR) in the body's glucose metabolism itself [37], and IR is also an essential mechanism of obesity and GDM.

Further analysis of the differences in species showed that there were significant differences in the abundance of eight strains in the NOR and GDM groups at the species level, whereas there were significant differences in the abundance of five strains in the G and LG groups. Additionally, the differential strains between the NOR and GDM groups are different from that between the G and LG groups, suggesting that there are differences in the long and short gestational gut microbes of GDM patients in the third trimester. Upon further analysis of the correlation between the different strains and the patient's blood sugar, the results revealed that the difference between the G and LG groups has a low correlation with the blood sugar. It could be that the amounts of the different strains are low and as such not enough to affect the blood sugar. The relative abundances of *Clostridium spiroforme*, *Eubacterium dolichum*, and *Ruminococcus gnavus* in the NOR and GDM groups were positively correlated with FBG, and *Pyramidobacter piscolens* was negatively correlated with FBG. Studies have shown that *Ruminococcus* can cause cells to absorb too much sugar, which can lead to obesity or overweight [38, 39]. However, some researchers believe that *Ruminococcus* can promote the metabolism of bile acids, which can bind with GBPAR1 and bile acid receptors (FXR) to help regulate the homeostasis of the intestinal flora and prevent intestinal microbes from releasing excessive lipopolysaccharides, which helps insulin to lower the blood sugar [40, 41]. The *Ruminococcus gnavus* species found in this paper is a species belonging to the genus *Ruminococcus*. The results of this study suggest that it is positively correlated with FBG during late pregnancy in patients with GDM.

Subsequently, changes in the specific functions caused by the changes in GDM intestinal microbes were analyzed. The different analyses showed that the intestinal microbes of the NOR and GDM groups were involved in the biosynthesis and metabolism, digestion, classification, and degradation of polysaccharides. There are differences in other biological effects, suggesting that there is a connection between the occurrence and development of GDM, and further molecular experiments are warranted to study the mechanism.

## 5. Conclusion

In a nutshell, with the development of 16S rDNA high-throughput sequencing, metagenomics, metabonomics, and other technologies, the research on intestinal flora and GDM has gradually deepened. Intestinal flora and metabolites have passed various pathogenic factors, inducing low-grade chronic inflammation and endotoxemia, causing IR, changing the pathway of bile acid metabolism, etc., comprehensively affecting the occurrence and development of GDM. The research on intestinal flora might adjust the dietary structure, prebiotics or probiotic preparations, and other programs for the treatment of GDM to realize the early prevention of GDM and personalized treatment and reduce the adverse pregnancy outcome for mothers and children. With further in-depth research on the relationship between gestational diabetes and the intestinal flora, it is believed that in the near future, beneficial bacteria can be supplemented to prevent and treat gestational diabetes, which is of great significance in promoting mother and child health and reducing the occurrence of diabetes.

## Figures and Tables

**Table 1:** Comparison of general conditions and biochemical indicators between the two groups of pregnant women (*x* ± *s*).

	NOR (*n*, 29)	GDM (*n*, 23)	G (*n*, 11)	LG (*n*, 12)
Age (years)	29.00 ± 1.88	29.80 ± 2.19	29.64 ± 2.29	29.67 ± 2.19
Gestational week (weeks)	36.10 ± 4.03	35.26 ± 3.65	32.03 ± 2.56	38.23 ± 1.35^∗^
Prepregnancy BMI (kg/m^2^)	21.39 ± 1.37	23.64 ± 1.36^∗^	23.62 ± 1.39	23.65 ± 1.39
FBG (mmol/L)	4.44 ± 0.42	5.29 ± 0.58^∗^	5.22 ± 0.76	5.36 ± 0.38
2 h blood glucose (mmol/L)	6.60 ± 0.73	9.30 ± 1.11^∗^	9.23 ± 1.31	9.37 ± 0.94
HbA1c (%)	5.05 ± 0.46	5.48 ± 0.21^∗^	5.53 ± 0.22	5.44 ± 0.20
TG (mmol/L)	2.11 ± 0.687	3.09 ± 1.20^∗^	3.36 ± 1.36	2.85 ± 1.03
CHOL (mmol/L)	4.96 ± 0.56	5.78 ± 0.79^∗^	5.86 ± 0.76	5.70 ± 0.85
HDL (mmol/L)	2.10 ± 0.34	1.81 ± 0.29^∗^	1.73 ± 0.25	1.88 ± 0.32
LDL (mmol/L)	2.69 ± 0.36	3.07 ± 0.54^∗^	3.10 ± 0.64	3.04 ± 0.47

^∗^ *P* < 0.05.

**Table 2:** NOR group vs. GDM group difference bacteria analysis at the species level.

Strain	NOR group (*n*, 29)	GDM group (*n*, 23)	*P*
Blautia_producta	0.12 ± 0.28	0.23 ± 0.30	0.01
Clostridium_spiroforme	0.002 ± 0.009	0.01 ± 0.03	0.004
Collinsella_aerofaciens	0.02 ± 0.03	0.06 ± 0.08	0.04
Coprococcus_catus	0.05 ± 0.08	0.13 ± 0.16	0.03
Eubacterium_dolichum	0.002 ± 0.003	0.02 ± 0.03	0.02
Pyramidobacter_piscolens	0.001 ± 0.003	0	0.01
Ruminococcus_callidus	0.09 ± 0.12	0.42 ± 0.57	0.03
Ruminococcus_gnavus	0.17 ± 0.13	0.66 ± 0.88	0.003

## Data Availability

The data used to support the findings of this study are available from the corresponding author upon request.

## Abbreviations

GDM: Gestational diabetes mellitus
OGTT: Oral glucose tolerance test
rRNA: Ribosomal RNA
FBG: Fasting blood glucose
OTU: Operational taxonomic unit
LEfSe: Linear discriminant analysis effect size
PLS-DA: Partial least squares discrimination analysis
TC: Total cholesterol
TG: Triacylglycerol
HDL-C: High-density lipoprotein cholesterol
LDL-C: Low-density lipoprotein cholesterol.
